# Address before the Hahnemann Club of Terre Haute

**Published:** 1890-02

**Authors:** W. H. Baker


### ADDRESS BEFORE THE HAHNEMANN CLUB OF TERRE HAUTE.

By W. H. Baker, M. D.

*Mr. President and members of the Hahnemann Club:*

To-night we begin the study of the *Organon*, one of the objects of our organization; a book that has fallen into disuse in one faction of our school, but which the history of medicine will prove has done more to bring order out of chaos, to place medicine on a firm foundation than any work published since the time of Hippocrates. It has revolutionized old physic and is an unerring guide for the cure of all ills of humanity. Each generation will have cause to be thankful that its author lived. Hahnemann, like all men who have proclaimed some great truth to the world, suffered persecution, but he lived to see the success of his discovery, and, to-day, thousands can testify to the truth of the law of similars: a law of cure that will go down through all the ages, giving to humanity the relief that it has been crying for in the centuries that have passed.

If we compare the strict followers of the *Organon* with the whole number of the practitioners of medicine, we will find them in the minority, but they are the vanguard of this army. Popular facts will come and go, but the truth of Homœopathy will live, and its followers increase as the world grows older and learns. Hahnemann never concluded on theory or speculation, but always on facts, and the doctrines contained in the *Organon* were only reached after years of thought and experience. He refused to accept Cullen's explanation of the curative power of Cinchona bark in curing chills and fever, and the great truth that unfolded itself to him while making that memorable translation you are all familiar with. He formulated the principles of Homœopathy, and we find them in the book we meet to study. On them our school must stand or fall; every student of medicine must follow these principles or he is anything but a homœopath. If we believe in the teachings of the *Organon*, let us follow it faithfully, and not run after strange gods; study its truths carefully and then put them into practice; we will find it a painstaking labor, but success will follow our efforts. Let us not read it as a curiosity, casting aside its precepts when meeting disease, but apply it, test it, and I feel sure we will see its beauty and efficacy as all who have followed its teachings. Hering, Bœnninghausen, Gross, Farrington, Lippe, and many others never found it wanting in teaching them the way to cure disease.

We may all meet cases where it seems to fail, but let us be careful in condemning, for the blame will be in ourselves, in our ignorance in applying it. We as a Society have a work to do here, in placing Homœopathy before the people in its true light, in demonstrating that pure Homœopathy is as far in advance of old physic with all its boasted progress as the electric light is in advance of the tallow dip, but let us be consistent and practice what we profess, and not make it possible for the dominant school to point us out as frauds. It we practice Homœopathy, we must follow the *Organon*, if we do not accept its teachings we should be honest and drop its name.

To prove one remedy carefully would be a good year's work for us. We can all help to verify the symptoms of our materia medica, perfecting in this way our means of applying the law of similars.

Bœnninghausen remarked to a brother physician that he had read the *Organon* fifteen times, and on each occasion he always acquired something new and valuable. Almost every page is rich in precept, and the student is constantly reminded that the only thing is the totality of the symptoms, the sole thing, in fact, which the physician has to take note of in every case of disease. The whole of the perceptible signs and symptoms which we can observe, expressing themselves through sensation and function, must be the sole indication to guide us in the choice of a curative remedy. No doubt we, too, will receive new light as we meet and discuss each paragraph how to fulfill "the highest and only calling of a physician, the restoration of health to the sick."

In the second paragraph we have described the highest ideal of a cure, which we are striving for, and I believe this can only be reached by a strict application of the principles laid down in the *Organon*.

The third paragraph teaches us that we must individualize each case of disease, also individualize the remedy in curing it. Empiricism and routine prescribing have no place in Homœopathy; the aggregate symptoms make up the individuality of each case. Every remedy has its own distinctive character, and we must find the one most like our case before we can reach "the highest aim of healing, the speedy, gentle, and permanent restoration of health, or alleviation and obliteration of disease in its entire extent, in the shortest, most reliable, and safest manner, according to clearly intelligible reasons."

When we have found the proper remedy, it is not all we have to know to secure a permanent recovery. There are numerous impediments which have multiplied since what is called civilization advances, and must be discovered and removed. Sewer gas that finds its way in through permanent washstands and closets of our modern apartments; decomposing matter in the cellar; the dry air of a furnace which retards the recovery from pneumonia and bronchitis. Many others might be mentioned but would make our paper too long.

In the third paragraph, we have the first intimation of a law of cure that we must follow if we would become true healers. There is also another point mentioned here that has caused much controversy, and one of the reasons of the division that exists in our school—viz., the preparation and the proper dose, and the proper time of its repetition. The *Organon* teaches *similia similibus curantur*, the single remedy and the minimum dose, and will not admit of any deviation.

Pathology can never furnish a true guide for a curative prescription. Persons attacked by the same disease do not suffer alike. Hahnemann was the first one to call attention to this fact, and the necessity of observing the totality of symptoms, "this outwardly reflected image of the inner nature of of the disease—*i. e.*, of the suffering vital force—must be the chief or only means of the disease to make known the remedy necessary for its cure."

The *Organon* points out how we are to examine the sick, how to acquire a knowledge of drugs, and the proper use of them in healing.

This is an age of progress, and if we take a review of medicine we find the stamp of progress here. We all rejoice in the advances made in surgery, pathology, and hygiene, but search the medical literature of the old school for a guide in therapeutics and you will find empiricism as dominant as ever—sad to know that some members of our school are filling menial positions in the same boat. Our main object as physicians is to cure disease, and we have an unerring guide in the law of similars, universal in its application, and any deviation is fallacious, unscientific, and empirical, often attended with fatal results.

Hahnemann says, "Imitate my mode of practice accurately and carefully, as pointed out in the *Organon* on chronic diseases, and you will find it confirmed at every step. Take one case of disease after another, note down all of its perceptible symptoms in the special manner pointed out in the *Organon*, then, guided by the characteristic and striking symptoms, select the appropriate remedy, and administer it in the smallest dose, according to the strict rules and observances pointed out in the *Organon*, and if it does not afford speedy, gentle, lasting help, publish the failure to the world, and the doctrine of Homœopathy shall stand abashed."

